# Serum Galectin-3 Level Is Positively Associated with Endothelial Dysfunction in Patients with Chronic Kidney Disease Stage 3 to 5

**DOI:** 10.3390/toxins13080532

**Published:** 2021-07-29

**Authors:** Bang-Gee Hsu, Chih-Hsien Wang, Yu-Hsien Lai, Jen-Pi Tsai

**Affiliations:** 1 School of Medicine, Tzu Chi University, Hualien 97004, Taiwan; gee.lily@msa.hinet.net (B.-G.H.); wangch33@gmail.com (C.-H.W.); hsienhsien@gmail.com (Y.-H.L.); 2 Division of Nephrology, Hualien Tzu Chi Hospital, Buddhist Tzu Chi Medical Foundation, Hualien 97004, Taiwan; 3 Division of Nephrology, Department of Internal Medicine, Dalin Tzu Chi Hospital, Buddhist Tzu Chi Medical Foundation, Chiayi 62247, Taiwan

**Keywords:** chronic kidney disease, C-reactive protein, endothelial function, galectin-3, vascular reactivity index

## Abstract

Galectin-3, which is a novel biomarker of cardiovascular stress and related to inflammation, could predict adverse cardiovascular events. However, its relationship with endothelial function in patients with chronic kidney disease (CKD) remains inconclusive. This study aimed to investigate the association between serum galectin-3 levels and endothelial function in patients with stages 3–5 CKD. Fasting blood samples were obtained from 130 patients. Serum galectin-3 levels were determined using the enzyme-linked immunosorbent assay. The endothelial function, demonstrated as a vascular reactivity index (VRI), was measured noninvasively through digital thermal monitoring test. Then, we sorted the patients into poor, intermediate, and good vascular reactivity (VRI < 1.0, 1.0 ≤ VRI < 2.0, and VRI ≥ 2.0), accounting for 24 (18.5%), 44 (33.8%), and 62 (47.7%) patients, respectively. As the VRI decreased, the serum galectin-3 and C-reactive protein (CRP) levels significantly increased. The galectin-3 value positively correlated with the CRP value but negatively correlated with estimated glomerular filtration rate. In multivariable stepwise linear regression analysis, serum log-transformed galectin-3 level and log-transformed CRP were significantly negatively associated with VRI values. Therefore, galectin-3 together with CRP is associated with VRI values and is a potential endothelial function modulator and a valuable biomarker of endothelial dysfunction in patients with CKD.

## 1. Introduction

Over the past few decades, as the number of patients with chronic kidney disease (CKD) progressively increased, the number of adverse cardiovascular (CV) outcomes also increased; thus, multiple studies have been concentrating on interventions against cardiovascular disease (CVD) associated with traditional [1,2,3] as well as CKD-specific factors such as inflammation, abnormal bone and mineral metabolism, and endothelial damage or dysfunction [4,5]. All of these risk factors could contribute to atherosclerosis, cardiomyopathy, and CV complications. In particular, endothelial dysfunction, which is the very basis of atherosclerosis, parallels with CKD stages along with an increased risk for CV complications [6,7,8]. In addition to traditional factors such as diabetes mellitus (DM), hyperlipidemia, and hypertension (HTN), uremia-specific factors such as inflammation and oxidative stress can modulate the process of endothelial dysfunction [9,10,11]. As a well-known factor in regulating cell growth, proliferation, and inflammation, galectin-3 was recently shown as a novel biomarker correlated with CKD progression as well as clinical adverse outcomes [12,13].

Galectin-3 is a novel 35 kDa, soluble β-galactoside-binding lectin expressed by epithelial cells, endothelial cells, and macrophages, with multiple biological abilities, including cell proliferation, differentiation, growth, and inflammation; it has also been related to cardiac fibrosis and heart failure and even long-term adverse prognosis of the general population [14] and patients with chronic heart failure [14,15], DM [16], or CKD [17,18]. Particularly, plasma galectin-3 correlates with a lower or even rapidly declining estimated glomerular filtration rate (eGFR) [13,19]. Furthermore, galectin-3 is independently associated with pulse wave velocity (PWV), carotid intima-media thickness, and systemic vascular resistance [20,21,22]. It also mediates the process of endothelial dysfunction [23,24]. As a result, the incidence of CVD increases, indicating that galectin-3 participates in CVD development.

Moreover, endothelial dysfunction is linked to adverse outcomes in patients with CKD [6,25]. Given that galectin-3 is involved in endothelial dysfunction, we aimed to examine the association between serum galectin-3 levels and endothelial function and the possible clinical risk factors for endothelial dysfunction measured by a digital thermal monitoring (DTM) test in patients with CKD.

## 2. Results

Of the 130 patients with CKD, 24 (18.5%), 44 (33.8%), and 62 (47.7%) had poor, intermediate, and good vascular reactivity index (VRI), respectively (Table 1). Age, gender, DM, HTN, and biochemical analyses including serum blood urea nitrogen (BUN), creatinine, lipid profiles, and fasting blood sugar showed no differences between these three patient groups. However, as the VRI decreased, the levels of serum galectin-3 (*p* < 0.001) and C-reactive protein (CRP, *p* = 0.001) significantly increased.

**Figure 1:**
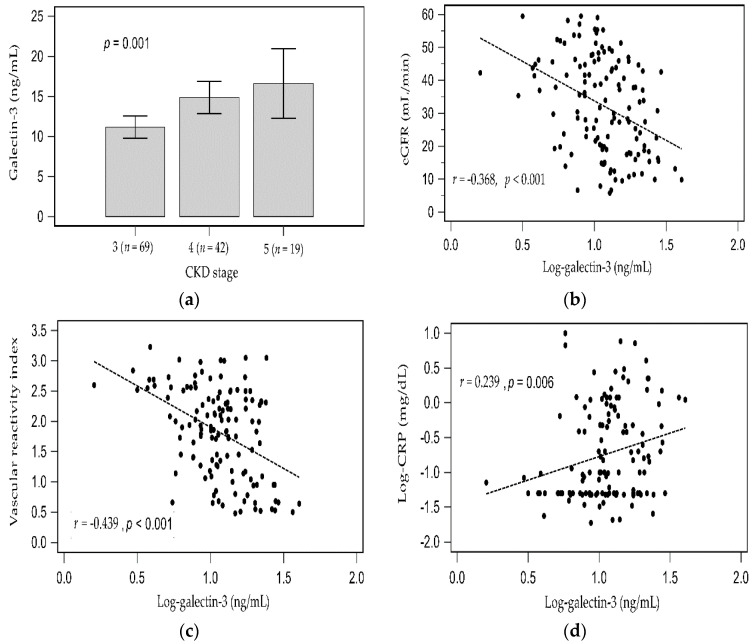
Difference of galectin-3 among CKD stage 3 to 5 analyzed by Kruskal–Wallis analysis (**a**), and simple linear regression analysis between (**b**) Log-galectin-3 and eGFR; (**c**) Log-galectin-3 and vascular reactivity index; (**d**) Log-galectin-3 and Log-CRP.

As the CKD stage progressed, the serum galectin-3 levels elevated (Figure 1a). In simple linear regression analysis, the log-transformed galectin-3 levels were weakly positively associated with log-transformed CRP (*r* = 0.239, *p* = 0.006) but negatively associated with eGFR (*r* = −0.368, *p* < 0.001) and VRI (*r* = −0.439, *p* < 0.001). Two-dimensional scattered plots of log-galectin-3 values with eGFR level, VRI, and serum log-CRP level among patients with CKD are presented as Figure 1b−d, respectively.

In a simple linear regression analysis, the log-transformed levels of BUN (*r* = −0.222, *p* = 0.011), creatinine (*r* = −0.191, *p* = 0.030), CRP (*r* = −0.410, *p* < 0.001), and galectin-3 (*r* = −0.439, *p* < 0.001) negatively correlated with the VRI values (Table 2). After adjusting for confounders (adapted factors were age, log-BUN, log-Creatinine, eGFR, log-CRP, and log- galectin-3), the multivariable stepwise linear regression analysis revealed that the log-transformed levels of CRP (β = −0.324, adjusted R^2^ change = 0.094; *p* < 0.001) and serum galectin-3 (β = −0.362, adjusted R^2^ change = 0.186; *p* < 0.001) were significantly and independently negatively associated with the VRI values (Table 2). The clinical variables of the CKD patients divided by CKD-level stratified statistics models as shown in Supplementary Table S1 (CKD stage 3), Table S2 (CKD stage 4), and Table S3 (CKD stage 5). After multivariable stepwise linear regression analysis, serum galectin-3 were also significantly and independently negatively associated with the VRI values in CKD-level stratified statistics models.

## 3. Discussion

This study showed that CRP and galectin-3 levels were negatively associated with VRI values measured by DTM among patients with CKD. After adjustment for confounders, the VRI values were independently negatively associated with CRP and galectin-3 in patients with CKD.

Traditional risk factors such as old age, HTN, and DM could cause atherosclerosis, cardiomyopathy, and CV complications in patients with CKD [4,5]. Endothelial dysfunction, which is another CV risk factor, as well as inflammation, is reportedly an independent and incremental predictor of coronary artery disease (CAD) or left ventricular mass index in patients with CKD [6,7,8]. This factor has also shown to be abnormal in such patients [9]. Using noninvasive modalities such as reactive hyperemia peripheral arterial tonometry and brachial artery ultrasound to evaluate endothelial function could predict the incidence of CAD and CV events in patients with CKD or peripheral arterial disease [6,25]. Furthermore, multiple biomarkers are linked to CKD-specific factors such as inflammation and oxidative stress that could lead to the abnormal regulation of endothelial function in predialysis patients with CKD [8,11]. A previous study reported that CRP is significantly negatively associated with brachial artery flow-mediated dilatation but positively associated with carotid artery intima-media thickness in CKD cases [11]. In the current study, we assessed endothelial function noninvasively and found that it was significantly associated with elevated CRP levels in patients with CKD.

Being ubiquitously expressed in epithelial cells, endothelial cells, and macrophages, galectin-3 regulates several types of inflammatory cells to promote fibrosis and inflammation in the kidney, heart, and vasculatures, resulting in long-term adverse effects [12,14,15,16,17]. As the galectin-3 level increases, all-cause mortality also increases by 1.1- to 2.17-fold in patients with acute or chronic heart failure, DM, or CKD and even the general population [14,15,16,17]. The renal role of galectin-3 is context-dependent. For instance, its expression is intensely upregulated in acute kidney disease [26]. It also prevents chronic renal tubular injury through inhibiting renal tubular apoptosis as well as modulating extracellular matrix remodeling, thereby attenuating fibrosis [27]. However, if the injury is persistent or repetitive, galectin-3 might promote inflammation and fibrosis [28], and the severity of renal fibrosis and tubular injury of renal transplant also depends on galectin-3 expression [29]. Galectin-3 levels strongly correlated with eGFR decline in the Ludwigshafen Risk and Cardiovascular Health study and were even more markedly elevated in the German Diabetes Mellitus Dialysis study; it could also identify individuals at risk of developing CKD or rapidly losing renal function over time in the general population and patients with CKD or chronic systolic heart failure [12,13,18,19]. Similarly, the present study revealed that galectin-3 was significantly associated with renal function decline and positively associated with elevated CRP values. In a mouse study, the downregulation of galectin-3 expression by treatment with modified citrus pectin lessened renal fibrosis, inflammation, and apoptosis [30]. Therefore, galectin-3 could predict adverse CV outcomes independently. Although the role of galectin-3 played in the progression or preservation of renal function remains inconclusive, galectin-3 might be a surrogate for kidney function and a promising biomarker targeting CKD progression.

In patients with rheumatoid arthritis, galectin-3 had a markedly positive association with arterial stiffness and atherosclerosis presented as increased PWV, carotid artery intima-media thickness, systemic vascular resistance, pulse pressure, and decreased total arterial compliance [20]. In addition, galectin-3 positively correlated with PWV in patients with chronic heart failure and hemodialysis after adjustment for confounders [21,22]. Overall, galectin-3 might have a role in cross-talk between vascular stiffness or remodeling and myocardial remodeling and mediate the process of endothelial dysfunction [23,24]; when both mechanisms occur, CVD develops. Endothelial dysfunction is reportedly caused by several mechanisms, including inflammation, dysregulated vascular remodeling, and vascular growth; in atherosclerosis, galectin-3 induces endothelial dysfunction [23,24,31]. Furthermore, galectin-3 together with oxidized low-density lipoprotein promotes the expression of lectin-like oxidized low-density lipoprotein receptor-1 (LOX-1), resulting in the enhanced generation of reactive oxygen species (ROS) and inflammatory responses through the LOX-1/ROS/NF-kB or integrin β1/RhoA/JNK signaling pathway; ultimately, these mechanisms can cause increased cytotoxicity of endothelial cells and promote atherosclerosis progression [23,24]. Another study showed that galectin-3 expression was markedly increased in a human atherosclerotic plaque and ApoE knockout mice fed with a high-fat diet; in an in vitro study, galectin-3 enhanced inflammatory markers such as CC chemokines through the macrophages [31]. Together with these studies showing that galectin-3 could be an indicator of vascular dysfunction and be associated with the inflammatory response of several diseases, our study showed that galectin-3 and CRP were independently associated with VRI and that galectin-3 might modulate endothelial dysfunction through the regulation of inflammatory pathways in patients with CKD.

However, this study has limitations such as the single-center, cross-sectional design and the small sample size. Thus, the causal relationship between serum galectin-3 levels and endothelial function should be confirmed by a longitudinal study with more patients.

## 4. Conclusions

In conclusion, together with CRP, galectin-3 was independently associated with endothelial dysfunction in patients with CKD.

## 5. Materials and Methods

### 5.1. Participants, Anthropometric Analysis, and Biochemical Investigations

We enrolled 130 patients with CKD admitted at Tzu Chi medical center in Hualien, Taiwan between January and December 2016. We reviewed their medical records to determine the underlying chronic diseases, including HTN and DM. Those patients with acute infection, acute CVD, heart failure, or malignancy were excluded. Prior to the study, we obtained informed consent from each eligible patient. We measured their body weight, body height, and blood pressure (BP). The levels of albumin, BUN, creatinine, fasting glucose, total cholesterol, triglycerides, low-density lipoprotein cholesterol, CRP, calcium, and phosphorus in their fasting blood samples were examined using an auto-analyzer (Siemens Advia 1800, Siemens Healthcare GmbH, Henkestr, Germany). Moreover, we measured their serum galectin-3 concentrations by using a commercially available enzyme-linked immunosorbent assay (RayBiotech, Peachtree Corners, GA, USA). Body mass index was calculated as the weight in kilograms divided by the height in meters squared. The eGFR was calculated using the Chronic Kidney Disease Epidemiology Collaboration equation [4]. Furthermore, the Research Ethics Committee of Hualien Tzu Chi Hospital in Buddhist Tzu Chi Medical Foundation approved this study (IRB103-136-B).

### 5.2. Endothelial Function Measurements

The endothelial function of all participants was measured using an FDA-approved device (Endothelix Inc., Houston, TX, USA) with DTM method, with BP cuffs and skin temperature sensors placed on both upper arms and index fingers, respectively [32,33]. In performing the DTM method, we first stably positioned both hands for 3 min, inflated the BP cuff to 50 mmHg greater than the systolic BP, and finally, deflated the BP cuff in 5 min. After BP cuff release, blood flowed into the forearm and hand, with the fingertips manifesting temperature rebound, which is proportional to reactive hyperemia. Using the VENDYS software, we measured the maximum difference between the observed temperature rebound curve and the zero-reactivity curve during the reactive hyperemia period to determine the VRI. According to the examination, participants were sorted into those with VRI < 1.0, 1.0 to <2.0, or ≥2.0 and defined as poor, intermediate, or good VRI, respectively [34].

### 5.3. Statistical Analysis

Continuous variables were tested using the Kolmogorov–Smirnov test. Nonnormally distributed variables such as triglyceride, BUN, creatinine, CRP, and galectin-3 were then log-transformed. These variables, which are expressed as means ± standard deviation or medians with interquartile ranges (IQRs), were analyzed by Kruskal–Wallis analysis or one-way analysis of variance (ANOVA) accordingly. Categorical variables were expressed as the number of patients and analyzed by χ^2^ test. Variables such as eGFR, VRI, and log-CRP, which correlated with log-galectin-3, were evaluated by simple linear regression analysis. Simple linear regression and multivariable stepwise linear regression analysis were applied to evaluate the association between VRI and clinical and biochemical variables. All statistical data were analyzed using the SPSS for Windows (version 19.0; SPSS Inc., Chicago, IL, USA), and *p* values < 0.05 were considered statistically significant.

## Figures and Tables

**Table 1:** Clinical characteristics according different vascular reactivity index by digital thermal monitoring of the 130 chronic kidney disease patients.

Characteristics	All Patients (*n* = 130)	Good Vascular Reactivity (*n* = 62)	Intermediate Vascular Reactivity (*n* = 44)	Poor Vascular Reactivity (*n* = 24)	*p* Value
Age (years)	66.05 ± 12.28	64.06 ± 11.00	68.32 ± 13.29	67.00 ± 13.14	0.196
Height (cm)	159.33 ± 8.66	159.95 ± 8.52	158.47 ± 9.29	159.31 ± 8.04	0.690
Body weight (kg)	68.24 ± 13.90	68.96 ± 12.90	68.45 ± 15.71	66.00 ± 13.19	0.674
Body mass index (kg/m^2^)	26.79 ± 4.52	26.90 ± 4.43	27.13 ± 4.83	25.90 ± 4.23	0.549
Vascular reactivity index	1.82 ± 0.74	2.46 ± 0.31	1.53 ± 0.31	0.69 ± 0.15	<0.001 *
Systolic blood pressure (mmHg)	135.78 ± 16.22	135.08 ± 14.85	137.14 ± 16.97	135.08 ± 18.62	0.794
Diastolic blood pressure (mmHg)	76.37 ± 11.15	77.81 ± 9.62	74.36 ± 11.60	76.33 ± 13.68	0.296
Albumin (g/dL)	4.23 ± 0.32	4.27 ± 0.32	4.17 ± 0.26	4.24 ± 0.42	0.290
Total cholesterol (mg/dL)	154.72 ± 31.98	156.82 ± 29.94	152.25 ± 35.19	153.79 ± 31.91	0.760
Triglyceride (mg/dL)	122.50 (90.00–172.25)	122.50 (93.75–185.50)	115.50 (87.75–152.25)	124.50 (88.25–181.50)	0.620
LDL-C (mg/dL)	80.28 ± 26.79	82.73 ± 26.31	77.68 ± 29.23	78.75 ± 23.71	0.607
Fasting glucose (mg/dL)	106.50 (91.00–128.50)	110.50 (94.00–130.50)	100.00 (89.00–123.75)	111.50 (91.00–132.25)	0.395
Blood urea nitrogen (mg/dL)	32.00 (24.00–49.00)	29.00 (24.00–42.25)	35.00 (24.00–55.50)	44.00 (24.25–56.50)	0.125
Creatinine (mg/dL)	1.90 (1.50–3.23)	1.90 (1.50–2.83)	1.95 (1.50–3.83)	2.55 (1.60–3.50)	0.177
eGFR (mL/min)	32.32 ± 14.75	34.92 ± 14.28	30.77 ± 15.65	28.47 ± 13.53	0.132
Total calcium (mg/dL)	9.11 ± 0.52	9.18 ± 0.54	9.02 ± 0.52	9.12 ± 0.49	0.340
Phosphorus (mg/dL)	3.65 ± 0.67	3.71 ± 0.69	3.65 ± 0.74	3.52 ± 0.48	0.507
Galectin-3 (ng/mL)	11.62 (8.55–17.34)	10.99 (6.82–14.07)	10.93 (8.60–14.10)	19.93 (12.77–27.38)	<0.001 *
C-reactive protein (mg/dL)	0.10 (0.05–0.66)	0.05 (0.05–0.47)	0.15 (0.05–0.67)	0.46 (0.21–1.28)	0.001 *
Female, *n* (%)	52 (40.0)	26 (41.9)	17 (38.6)	9 (37.5)	0.908
Diabetes mellitus, *n* (%)	61 (46.9)	29 (46.8)	20 (45.5)	12 (50.0)	0.937
Hypertension, *n* (%)	106 (81.5)	48 (77.4)	37 (84.1)	21 (87.5)	0.483
CKD stage 3, *n* (%)	69 (53.1)	37 (59.7)	22 (50.0)	10 (41.7)	0.078
CKD stage 4, *n* (%)	42 (32.3)	20 (32.3)	11 (25.0)	11 (45.8)	
CKD stage 5, *n* (%)	19 (14.6)	5 (8.1)	11 (25.0)	3 (12.5)	

Values for continuous variables given as means ± standard deviation and test by one-way analysis of variance; variables not normally distributed given as medians and interquartile range and test by Kruskal–Wallis analysis; values are presented as number (%) and analysis after analysis by the chi-square test. LDL-C, low-density lipoprotein cholesterol; eGFR, estimated glomerular filtration rate; CKD, chronic kidney disease. * *p* < 0.05 was considered statistically significant.

**Table 2:** Correlation of vascular reactivity index levels and clinical variables by simple or multivariable linear analyses among 130 chronic kidney disease patients.

Variables	Vascular Reactivity Index				
	Simple Regression		Multivariate Regression		
	*r*	*p*-Value	Beta	Adjusted R^2^ Change	*p*-Value
Female	0.093	0.295	—	—	—
Diabetes mellitus	−0.051	0.567	—	—	—
Hypertension	−0.077	0.384	—	—	—
Age (years)	−0.148	0.093	—	—	—
Height (cm)	−0.020	0.824	—	—	—
Body weight (kg)	0.055	0.532	—	—	—
Body mass index (kg/m^2^)	0.086	0.331	—	—	—
Systolic blood pressure (mmHg)	−0.020	0.824	—	—	—
Diastolic blood pressure (mmHg)	0.111	0.207	—	—	—
Albumin (g/dL)	0.024	0.789	—	—	—
Total cholesterol (mg/dL)	0.099	0.265	—	—	—
Log-triglyceride (mg/dL)	0.054	0.542	—	—	—
LDL-C (mg/dL)	0.067	0.449	—	—	—
Log-glucose (mg/dL)	−0.092	0.296	—	—	—
Log-BUN (mg/dL)	−0.222	0.011 *	—	—	—
Log-creatinine (mg/dL)	−0.191	0.030 *	—	—	—
eGFR (mL/min)	0.165	0.061	—	—	—
Total calcium (mg/dL)	0.077	0.381	—	—	—
Phosphorus (mg/dL)	0.097	0.273	—	—	—
Log-CRP (mg/dL)	−0.410	<0.001 *	−0.324	0.094	<0.001 *
Log-galectin-3 (ng/mL)	−0.439	<0.001 *	−0.362	0.186	<0.001 *

Data of galectin-3, triglyceride, glucose, blood urea nitrogen, creatinine, and C-reactive protein levels showed skewed distribution and, therefore, were log-transformed before analysis. Analysis of data was done using the simple linear regression analyses or multivariable stepwise linear regression analysis (adapted factors were age, log-BUN, log-Creatinine, eGFR, log-CRP, and log- galectin-3). LDL-C, low-density lipoprotein cholesterol; BUN, blood urea nitrogen; eGFR, estimated glomerular filtration rate; CRP, C-reactive protein. * *p* < 0.05 was considered statistically significant.

## Data Availability

The data presented in this study are available on request from the corresponding author.

## Supplementary Materials

The following are available online at https://www.mdpi.com/article/10.3390/toxins13080532/s1, Table S1: Correlation of vascular reactivity index levels and clinical variables by simple or multivariable linear analyses among 69 patients with chronic kidney disease stage 3. Table S2: Correlation of vascular reactivity index levels and clinical variables by simple or multivariable linear analyses among 42 patients with chronic kidney disease stage 4. Table S3: Correlation of vascular reactivity index levels and clinical variables by simple or multivariable linear analyses among 19 patients with chronic kidney disease stage 5.
