# Supplementary material for: Serum Galectin-3 Level Is Positively Associated with Endothelial Dysfunction in Patients with Chronic Kidney Disease Stage 3 to 5
Source: Toxins (Basel). 2021 Jul 29;13(8):532. doi: 10.3390/toxins13080532 (PMC8402460; doi:10.3390/toxins13080532)
Supplement: Supplementary file 1 [file toxins-13-00532-s001.zip › toxins-1303995-Supplementary.pdf]

Supplementary Materials: Serum Galectin-3 Level Is Positively Associated with Endothelial Dysfunction in Patients with Chronic Kidney Disease stage 3 to 5

Bang-Gee Hsu, Chih-Hsien Wang, Yu-Hsien Lai and Jen-Pi Tsai

Table S1. Correlation of vascular reactivity index levels and clinical variables by simple or multivariable linear analyses among 69 patients with chronic kidney disease stage 3.

Variables	Vascular Reactivity Index				
	Simple Linear Regression		Multivariable Linear Regression		
	r	*p* Value	Beta	Adjusted R^2 change	*p* Value
Female	0.017	0.887	—	—	—
Diabetes mellitus	−0.054	0.661	—	—	—
Hypertension	−0.036	0.770	—	—	—
Age (years)	−0.270	0.025*	—	—	—
Height (cm)	0.099	0.420	—	—	—
Body weight (kg)	0.196	0.102	—	—	—
Body mass index (kg/m^2)	0.177	0.147	—	—	—
Systolic blood pressure (mmHg)	−0.122	0.318	—	—	—
Diastolic blood pressure (mmHg)	0.109	0.372	—	—	—
Albumin (g/dL)	−0.087	0.478	—	—	—
Total cholesterol (mg/dL)	0.069	0.573	—	—	—
Triglyceride (mg/dL)	−0.025	0.836	—	—	—
LDL-C (mg/dL)	0.133	0.277	—	—	—
Fasting glucose (mg/dL)	−0.020	0.872	—	—	—
BUN (mg/dL)	−0.161	0.188	—	—	—
Log-Creatinine (mg/dL)	−0.039	0.749	—	—	—
eGFR (mL/min)	0.087	0.475	—	—	—
Total calcium (mg/dL)	0.182	0.134	—	—	—
Phosphorus (mg/dL)	0.073	0.549	—	—	—
Log-CRP (mg/dL)	−0.480	<0.001*	−0.378	0.219	0.001*
Galectin-3 (ng/mL)	−0.466	<0.001*	−0.359	0.110	0.001*

Data of creatinine, and C-reactive protein levels showed skewed distribution, and therefore were log-transformed before analysis. Analysis of data was done using the simple linear regression analyses or multivariable stepwise linear regression analysis (adapted factors were age, log-CRP and galectin-3). LDL-C, low density lipoprotein cholesterol; BUN, blood urea nitrogen; eGFR, estimated glomerular filtration rate; CRP, C-reactive protein. *$p < 0.05$ was considered statistically significant.

Table S2. Correlation of vascular reactivity index levels and clinical variables by simple or multivariable linear analyses among 42 patients with chronic kidney disease stage 4.

Variables	Vascular Reactivity Index				
	Simple Linear Regression		Multivariable Linear Regression		
	r	*p* Value	Beta	Adjusted R^2 change	*p* Value

Female	0.183	0.245	—	—	—
Diabetes mellitus	−0.089	0.575	—	—	—
Hypertension	−0.110	0.488	—	—	—
Age (years)	−0.345	0.025*	—	—	—
Height (cm)	0.004	0.982	—	—	—
Body weight (kg)	0.094	0.553	—	—	—
Body mass index (kg/m²)	0.101	0.525	—	—	—
Systolic blood pressure (mmHg)	0.171	0.279	—	—	—
Diastolic blood pressure (mmHg)	0.238	0.128	—	—	—
Albumin (g/dL)	0.002	0.987	—	—	—
Total cholesterol (mg/dL)	0.171	0.280	—	—	—
Triglyceride (mg/dL)	0.189	0.230	—	—	—
LDL-C (mg/dL)	0.106	0.502	—	—	—
Fasting glucose (mg/dL)	−0.092	0.564	—	—	—
BUN (mg/dL)	−0.138	0.383	—	—	—
Creatinine (mg/dL)	−0.189	0.230	—	—	—
eGFR (mL/min)	0.109	0.493	—	—	—
Total calcium (mg/dL)	−0.215	0.172	—	—	—
Phosphorus (mg/dL)	0.304	0.050	—	—	—
Log-CRP (mg/dL)	−0.375	0.014*	−0.415	0.158	0.003*
Galectin-3 (ng/mL)	−0.410	0.007*	−0.448	0.148	0.001*

Data of C-reactive protein levels showed skewed distribution, and therefore were log-transformed before analysis. Analysis of data was done using the simple linear regression analyses or multivariable stepwise linear regression analysis (adapted factors were age, log-CRP and galectin-3). LDL-C, low density lipoprotein cholesterol; BUN, blood urea nitrogen; eGFR, estimated glomerular filtration rate; CRP, C-reactive protein. **p* < 0.05 was considered statistically significant.

Table S3. Correlation of vascular reactivity index levels and clinical variables by simple or multivariable linear analyses among 19 patients with chronic kidney disease stage 5.

**Variables**	**Vascular Reactivity Index**				
	**Simple Linear Regression**		**Multivariable Linear Regression**		
	r	*p* Value	Beta	Adjusted R^2 change	*p* Value
Female	0.326	0.173	—	—	—
Diabetes mellitus	0.059	0.812	—	—	—
Hypertension	−0.171	0.483	—	—	—
Age (years)	0.345	0.149	—	—	—
Height (cm)	−0.443	0.058	—	—	—
Body weight (kg)	−0.365	0.124	—	—	—
Body mass index (kg/m²)	−0.194	0.426	—	—	—
Systolic blood pressure (mmHg)	0.086	0.725	—	—	—
Diastolic blood pressure (mmHg)	−0.126	0.608	—	—	—
Albumin (g/dL)	0.118	0.630	—	—	—
Total cholesterol (mg/dL)	−0.165	0.501	—	—	—
Triglyceride (mg/dL)	0.036	0.883	—	—	—
LDL-C (mg/dL)	−0.402	0.088	—	—	—
Log-Glucose (mg/dL)	−0.339	0.155	—	—	—

BUN (mg/dL)	−0.390	0.099	—	—	—
Creatinine (mg/dL)	−0.302	0.209	—	—	—
eGFR (mL/min)	0.145	0.552	—	—	—
Total calcium (mg/dL)	0.261	0.280	—	—	—
Phosphorus (mg/dL)	0.103	0.674	—	—	—
CRP (mg/dL)	−0.509	0.026*	—	—	—
Galectin-3 (ng/mL)	−0.511	0.025*	−0.511	0.217	0.025*

Data of glucose levels showed skewed distribution, and therefore were log-transformed before analysis. Analysis of data was done using the simple linear regression analyses or multivariable stepwise linear regression analysis (adapted factors were CRP and galectin-3). LDL-C, low density lipoprotein cholesterol; BUN, blood urea nitrogen; eGFR, estimated glomerular filtration rate; CRP, C-reactive protein. *$p < 0.05$ was considered statistically significant.
